# Associations Between Natural Physiological and Supraphysiological Estradiol Levels and Stress Perception

**DOI:** 10.3389/fpsyg.2019.01296

**Published:** 2019-06-11

**Authors:** Brigitte Leeners, Tillmann H. C. Krüger, Kirsten Geraedts, Enrico Tronci, Toni Mancini, Marcel Egli, Susanna Röblitz, Lanja Saleh, Katharina Spanaus, Cordula Schippert, Yuanyuan Zhang, Fabian Ille

**Affiliations:** ^1^ Department of Reproductive Endocrinology, University Hospital Zurich, Zurich, Switzerland; ^2^ Department of Psychiatry, Social Psychiatry and Psychotherapy, Medical School Hannover, Hannover, Germany; ^3^ Department of Computer Science, University of Rome “La Sapienza”, Rome, Italy; ^4^ Centre of Competence in Aerospace Biomedical Science & Technology, Lucerne University of Applied Sciences and Arts, Lucerne, Switzerland; ^5^ Computational Biology Unit, Department of Informatics, University of Bergen, Bergen, Norway; ^6^ Institute of Clinical Chemistry, University Hospital Zurich, Zurich, Switzerland; ^7^ Department of Gynaecology and Obstetrics, Hannover Medical School, Hannover, Germany

**Keywords:** hormones, fertility treatment, menstrual cycle, stress, PSQ, cognitive bias, estrogen

## Abstract

Stress is a risk factor for impaired general, mental, and reproductive health. The role of physiological and supraphysiological estradiol concentrations in stress perception and stress processing is less well understood. We, therefore, conducted a prospective observational study to investigate the association between estradiol, stress perception, and stress-related cognitive performance within serial measurements either during the natural menstrual cycle or during fertility treatment, where estradiol levels are strongly above the physiological level of a natural cycle, and consequently, represent a good model to study dose-dependent effects of estradiol. Data from 44 women receiving *in vitro* fertilization (IVF) at the Department of Reproductive Endocrinology in Zurich, Switzerland was compared to data from 88 women with measurements during their natural menstrual cycle. The German version of the Perceived Stress Questionnaire (PSQ) and the Cognitive Bias Test (CBT), in which cognitive performance is tested under time stress were used to evaluate subjective and functional aspects of stress. Estradiol levels were investigated at four different time points during the menstrual cycle and at two different time points during a fertility treatment. Cycle phases were associated with PSQ worry and cognitive bias in normally cycling women, but different phases of fertility treatment were not associated with subjectively perceived stress and stress-related cognitive bias. PSQ lack of joy and PSQ demands related to CBT in women receiving fertility treatment but not in women with a normal menstrual cycle. Only strong changes of the estradiol level during fertility treatment were weakly associated with CBT, but not with subjectively experienced stress. Our research emphasizes the multidimensional character of stress and the necessity to adjust stress research to the complex nature of stress perception and processing. Infertility is associated with an increased psychological burden in patients. However, not all phases of the process to overcome infertility do significantly increase patient stress levels. Also, research on the psychological burden of infertility should consider that stress may vary during the different phases of fertility treatment.

**Clinical trial registration:** ClinicalTrials.gov # NCT02098668.

## Introduction

A variety of research results support an involvement of steroid hormones in the perception and processing of stress. The prevalence of affective disorders and stress-related disorders such as port-traumatic stress disorder (PTSD) is two to three times higher in women (Kessler et al., 2005; Tolin and Foa, 2006; Bromet et al., 2011; Zoladz and Diamond, 2013). These sex differences start at puberty and persist until menopause (Kessler et al., 1994; Zahn-Waxler et al., 2008).

In addition, the symptoms severity in different psychiatric diseases seems to vary with the cycle, with symptom improvement during the high-estradiol phase (Baca-Garcia et al., 2004; Davydov et al., 2005; Saunders and Hawton, 2006; Gonda et al., 2008). However, longitudinal measurements have demonstrated that in healthy women, the sex hormones do not consistently and robustly relate to negative affect or cognitive performance when assessed across two consecutive menstrual cycles (Hengartner et al., 2017; Leeners et al., 2017). In sight of such results, many of the associations interpreted as consequences of hormonal variations have to be attributed to methodological limitations.

Differences in stress system response, for example, a decreased cortisol response in the late follicular phase (Kirschbaum et al., 1999) have been reported to correlate with the menstrual cycle (Roca et al., 2005; Kajantie and Phillips, 2006). Normal ovarian hormone fluctuations seem to alter the impact of psychosocially stressful events, i.e., low estradiol levels have been reported to be associated with an increased vulnerability to psychosocial stress, for example, with an increased risk to develop PTSD (Glover et al., 2012; Albert et al., 2015). Estradiol receptors are located in brain areas that are important for the response to psychosocial stress (Love et al., 2010). In series of 12 women, brain structures involved in fear and arousal processing show attenuated responses to emotional stimuli during the late follicular phase of the menstrual cycle (Goldstein, 2005; Goldstein et al., 2010). Another small fMRI study suggested that low estradiol levels exaggerate the effect of psychosocial stress on brain activity (Albert et al., 2015); however, the small sample sizes in all three studies limit the reliability of these findings (Button et al., 2013; Turner et al., 2018). Women with higher estradiol levels appear to have less subjective distress and increased negative mood in response to a stress task when compared to women with lower estradiol levels (Albert et al., 2015).

A diagnosis of infertility is accompanied by increased stress levels, with psychological stress outweighing by far the stress associated with medical procedures (Cousineau and Domar, 2007). As stress is discussed to influence not only the success of fertility treatments but also the decision to adhere to medical support (Klonoff-Cohen et al., 2001; Klonoff-Cohen and Natarajan, 2004; Domar et al., 2015; Frederiksen et al., 2015), it is important to understand stress perception and stress processing in the context of infertility. Only few studies have investigated the effect of the estradiol level on stress perception and regulation or have compared subjective stress perception with a functional test (Holmes et al., 2002; Barker and Galea, 2010). Estradiol reaches particularly high levels at the end of a stimulation phase during fertility treatment. Therefore, this condition represents a good model to evaluate the dose-dependent effect of estradiol.

As stress seems to play a major role in women’s reproductive health, it is important to better understand associations between estradiol levels and subjective as well as objective stress-related cognitive measures in the context of a natural cycle but also in the context of fertility treatment. On this background, the aim of our study was to evaluate whether (1) the subjective perception of stress and the results from a cognitive test sensitive to stress vary throughout the menstrual cycle and during the different phases of fertility treatment, (2) whether stress perception and stress-related cognitive test measurements covary, and (3) whether estradiol levels are associated with stress perception and stress-related cognitive performance.

## Materials and Methods

### Study Design

We conducted a prospective observational study on the association of hormonal and psychological parameters collected within serial measurements, either during the natural menstrual cycle or during a fertility treatment. We chose to compare data from these two groups as estradiol reaches much higher levels (up to more than 10-fold) during fertility treatment than during a natural menstrual cycle, which represents a good model to investigate associations between hormonal and psychological parameters.

### Study Group

Data from 44 women receiving *in vitro* fertilization (IVF) at the Department of Reproductive Endocrinology, University Hospital Zurich, Switzerland was compared to measurements during natural cycles of 88 healthy women recruited at the University Hospital Zurich and the Medical School Hannover.

The measurements were performed at key time points of a fertility treatment, e.g., the end of the downregulation/preparation phase, where hormone values are lowest and at the end of the stimulation phase where estradiol values are highest.

In women monitored during their menstrual cycle, measurements were taken at the early follicular phase, the preovulatory phase, the midluteal phase, and shortly prior to expected menses. Further details of hormone measurements in women during their natural menstrual cycle have been previously reported (Hengartner et al., 2017; Leeners et al., 2017). Results of cognitive performance throughout two consecutive cycles have been described earlier (Leeners et al., 2017).

Women undergoing fertility treatments were subjected to the normal investigation of fertility disorders at the Department of Reproductive Endocrinology, University Hospital Zurich, Switzerland. A gynecological examination and transvaginal ultrasound were performed to determine the antral follicle count and uterine or adnexal abnormalities. The evaluation of hormones (LH, FSH, estradiol, anti-Mullerian hormone; testosterone, 17 hydroxyprogesterone, prolactin, thyroid stimulating hormone on specific indication) in the early follicular phase (day 2–5) served to investigate endocrinological disorders. A semen analyses was conducted in the male partners. Depending on the result of the semen analysis and eventual urological treatment, a hydrosonography of the uterine cavity, a hydro-contrast-sonography or a hysterosalpingography were performed to evaluate uterine and/or tubal pathology. Hepatitis B, C, HIV, and chlamydia infection was investigated in both partners. Within the initial patients’ history, relevant inclusion and exclusion criteria were evaluated, for example, linguistic capacity or medical conditions, which might influence cognitive performance such as psychiatric diseases. Premenstrual syndrome was also an exclusion criterion for study participation.

### Measurements of Stress

#### Perceived Stress Questionnaire

The Perceived Stress Questionnaire (PSQ) (Fliege et al., 2005) conceptualized by Levenstein et al. (1993) was used to evaluate subjectively perceived stress. The German version of the PSQ has been validated in a German sample (*N* = 650) (Fliege et al., 2001). In contrast to the original PSQ, only the factors worry, tension, joy, and demands could be confirmed. These scales are composed of five items each resulting in a total of 20 items that show good internal consistency with Cronbach’s *α* between 0.80 and 0.86. The scales “worries,” “tension,” and “joy” are estimated to represent the internal stress reaction, while the variable “demands” is considered to represent experiences toward external stressors. Construct validity has been evaluated by investigating associations with subjectively perceived quality of life as measured with the World Health Organization Quality of Life Questionnaires in its short-version (WHOQOL-Bref) and with social support as measured with a German questionnaire to evaluate social support (“Fragebogen zur Sozialen Unterstützung,” F-SOZU). A strong association with results from both questionnaires confirms high construct validity. External validity was supported by results from psychosomatic patients prior to therapy, women after abortion, and women after normal delivery. The German version of the PSQ seems to be sensitive to longitudinal changes of stress experiences as has been demonstrated by decreasing stress values during in-patients psychotherapy (Fliege et al., 2001). For each item, the study participants had to choose the most appropriate answer for the last 24 h between the options “rarely,” “sometimes,” “often,” and “mostly,” i.e., a numerical scale ranging from 1 to 4, where one is the least and 4 is the most.

#### Cognitive Bias Test

The Cognitive Bias Test (CBT) is a multiple choice procedure designed by Goldberg et al. (1994) as a bias (preference) to evaluate complex cognitive functions. The CBT entails designs characterized along five binary dimensions: shape (circle/square), color (red/blue), number (one/two identical components), size (large/small), and contour (outline/filled with a homogeneous color). Study participants have to rate similarity between two items. The items are on different levels of difficulty and presented twice in different vertical positions to the study participant. Thus, 32 stimuli can be generated, and a “similarity index” computed between any two stimuli, ranging from 5 (identical) to 0 (differing along all five dimensions). The “similarity indices” between targets and subject’s choices are summed across trials (Goldberg et al., 1994). In the present study, we used correct responses as the outcome, that is, higher scores on the CBT indicate better cognitive control. The decisions have to be taken under time stress, that is, after few seconds, noises and blinking color signals increase the pressure to take a decision. On this background, the cognitive bias test is considered to not only capture cognitive performance, but also functional reactions toward stress (Lehner et al., 1997; Yu, 2016).

### Ethics

This study followed the guidelines of the World Medical Association Declaration of Helsinki 1964, updated in October 2013 and was conducted after approval by the Cantonal Committee of Zürich, Switzerland. All participants provided a written informed consent for study participation. Women were compensated for their expenditures associated with the study participation. The study has been registered in clin.trial.gov (NCT02098668).

### Statistics

Estradiol levels were log-transformed for statistical analysis. Repeated measures of PSQ and CBT were examined using Generalized Estimating Equations (GEE). These statistical models were introduced to fit regression analyses that account for within-subject correlation, which is an inherent part of longitudinal studies that rely on repeated outcome measures (Zeger et al., 1988). GEE are considered state-of-the-art for longitudinal data analysis and superior to repeated measures ANOVA due to their psychometric properties (Ballinger, 2004; Gibbons et al., 2010). GEE use all available data and impute missing values under the assumption of Missing Completely at Random (MCAR). Because all PSQ dimensions and CBT scores were right skewed interval scales, we fitted all models with Gamma distribution and log link-function. The within-subject covariance was specified with the “unstructured” correlation type to avoid having any constraints on the covariance structure and a robust sandwich estimator was used to reduce the effects of outliers and influential observations. All analyses were conducted with SPSS version 24.

## Results

Altogether, 85 women were investigated during their natural menstrual cycle and 44 during a fertility treatment. Mean age in the first group was 30.2 ± 5.5 years (range 20–43) and 36.0 ± 3.4 years (range 28–44; *p* < 0.001) in women undergoing fertility treatment. In 13 women, fertility treatment was performed because of a mechanical problem, in 14 because of endometriosis in 10 because of polycystic ovary syndrome (PCOS), in 6 because of idiopathic sterility and in 34 women either because of male factor only or a reduced sperm quality in addition to the listed female indications. Some of the couples had several causes of infertility.

No differences in baseline estradiol levels between women in their natural cycle and women in fertility treatment nor between women receiving fertility treatment because of female indication only, combined male and female indication or male indication only was found.

### Variation of Perceived Stress Questionnaire and Cognitive Bias Test Across Time

PSQ worry varied significantly across the menstrual cycle (*p* = 0.001). The mean scores declined steadily across measurement occasion. A linear time trend was statistically confirmed using a polynomial contrast analysis (contrast estimate: −0.774, SE = 0.193; Wald *χ*^2^ = 16.069, df = 1, *p* < 0.001). PSQ tension, PSQ lack of joy, and PSQ demands remained stable across the cycle, whereas for cognitive bias, there was again evidence of significant change (*p* = 0.004). On a descriptive level, cognitive bias was highest during menstrual phase, declined markedly at pre-ovulatory phase and reached an intermediate plateau across both mid-luteal and pre-menstrual phase. According to a polynomial contrast analysis, the time trend was quadratic (contrast estimate: 0.623, SE = 0.224, Wald *χ*^2^ = 7.722, df = 1, *p* = 0.016). In women receiving fertility treatment, all PSQ scales as well as cognitive bias remained stable across time. Comparing the PSQ scales and cognitive bias between groups (controls versus fertility treatment) revealed no significant differences, as all confidence intervals overlapped. That is, control women and women receiving fertility treatment did not differ in both their perceived stress and cognitive bias.

### Covariation Between Perceived Stress Questionnaire and Cognitive Bias Test

Next, we examined whether perceived stress and cognitive bias covary across time. As indicated in Table 1, in the control group, PSQ scales were not associated with cognitive bias, indicating that, inter-individually, subjectively perceived stress does not correlate with cognitive bias. In contrast, in women receiving fertility treatment, there was a small negative association with PSQ lack of joy and PSQ demands. These findings indicate that, women who report both increased lack of joy and demands, have lower cognitive bias scores. Both effect sizes corresponded to approximately Pearson *r* = −0.2.

**Table 1:** Associations of perceived stress with cognitive bias across time.

	Cognitive bias		
	B	95% CI	p^*^
**PSQ worry**			
Control	0.009	−0.003; 0.020	0.137
Fertility treatment	−0.010	−0.020; 0.000	0.053
**PSQ tension**			
Control	0.006	−0.005; 0.017	0.250
Fertility treatment	−0.005	−0.015; 0.005	0.333
**PSQ lack of joy**			
Control	0.003	−0.006; 0.013	0.499
Fertility treatment	−0.017	−0.030; −0.004	0.009
**PSQ demands**			
Control	0.008	−0.001; 0.018	0.070
Fertility treatment	−0.020	−0.031; −0.009	0.001

* *Adjusted α = 0.01*.

### Covariation Between Perceived Stress Questionnaire, Cognitive Bias Test, and Estradiol

The log-transformed estradiol levels at different time points are presented in Table 2. The repeated associations of estradiol with PSQ scales and cognitive bias are indicated in Table 3. In the control group, there was a negative association between PSQ demands and estradiol levels corresponding to an effect size of approximately Pearson *r* = −0.1. That is, women who had higher estradiol levels over time reported slightly lower demands. However, this association did not reach statistical significance when adjusted for multiple testing (adjusted *α* = 0.01). In women receiving fertility treatment, estradiol was positively associated with cognitive bias, indicating that women with higher estradiol levels experienced more cognitive bias. The effect size corresponded to Pearson *r* = 0.2 and remained statistically significant after adjusting for multiple testing.

**Table 2:** Perceived stress and cognitive bias across time.

	Measurement occasion				Model effect
Measurements during menstrual cycle	T1 Menstrual phase	T2 Pre-ovulatory phase	T3 Mid-luteal phase	T4 Premenstrual phase	
Measurements during fertility treatment	End of down-regulation/preparation phase	End of stimulation phase			
	Mean (95% CI)	Mean (95% CI)	Mean (95% CI)	Mean (95% CI)	*p*
**PSQ worry**					
Control	9.7 (8.9–10.5)	9.1 (8.4–9.9)	8.9 (8.1–9.6)	8.6 (7.9–9.3)	0.001
Fertility treatment	8.9 (8.0–9.8)	8.8 (7.9–9.8)			0.809
**PSQ tension**					
Control	11.4 (10.7–12.1)	11.1 (10.4–11.8)	11.1 (10.5–11.8)	11.3 (10.6–12.0)	0.694
Fertility treatment	11.2 (10.2–12.2)	11.2 (10.2–12.4)			0.860
**PSQ lack of joy**					
Control	9.9 (9.3–10.6)	10.2 (9.5–10.9)	10.2 (9.6–10.9)	10.3 (9.6–11.0)	0.574
Fertility treatment	10.3 (9.5–11.2)	10.1 (9.1–11.1)			0.645
**PSQ demands**					
Control	11.7 (11.1–12.4)	11.0 (10.4–11.7)	10.8 (10.1–11.6)	11.1 (10.4–11.7)	0.093
Fertility treatment	10.3 (9.5–11.2)	10.4 (9.3–11.6)			0.831
**Cognitive bias**					
Control	7.4 (6.7–8.1)	6.2 (5.6–6.8)	6.5 (5.8–7.2)	6.5 (5.8–7.3)	0.004
Fertility treatment	7.1 (6.2–8.1)	7.2 (5.7–9.0)			0.902
**Estradiol (log)**					
Control	5.0 (4.9–5.1)	6.4 (6.3–6.6)	6.3 (6.2–6.4)	5.7 (5.5–5.8)	<0.001
Fertility treatment	3.4 (3.1–3.8)	8.2 (8.0–8.5)			<0.001

**Table 3:** Associations between E2 and stress.

	E2 levels		
	B	95% CI	p^*^
**PSQ worry**			
Control	−0.009	−0.056; 0.039	0.723
Fertility treatment	−0.008	−0.058; 0.043	0.767
**PSQ tension**			
Control	−0.023	−0.062; 0.015	0.236
Fertility treatment	0.018	−0.034; 0.071	0.491
**PSQ lack of joy**			
Control	0.003	−0.035; 0.042	0.864
Fertility treatment	0.015	−0.041; 0.071	0.603
**PSQ demands**			
Control	−0.054	−0.100; −0.008	0.021
Fertility treatment	0.018	−0.048; 0.083	0.600
**Cognitive bias**			
Control	−0.053	−0.123; 0.016	0.132
Fertility treatment	0.232	0.120; 0.345	<0.001

* *Adjusted α = 0.01*.

## Discussion

During the natural cycle, worries exhibit higher values during the menstrual period, and to a lesser degree during the pre-ovulatory phase. Experiences of tension, lack of joy and demands do not change with cycle phases. Also, in women receiving fertility treatment, no differences between various aspects of perceived stress could be demonstrated between the end of the downregulation/preparation phase and the end of the stimulation phase, where estradiol values are highest. In cycling women, stress-related cognitive bias was highest during the menstrual phase, while no differences were detected in the evaluated phases of a fertility treatment. During the natural cycle, no significant associations between estradiol levels and scores in the PSQ or the CBT could be demonstrated. In contrast, higher levels of estradiol as in fertility treatments were associated with increased stress-related cognitive bias; however, the effect size was rather small.

Interestingly, no significant differences in the perceived stress level could be demonstrated between women in their natural cycle and women receiving fertility treatment. Also, results in the PSQ as representing subjectively experienced stress and results from the CBT as a functional measurement did not correlate in women with a natural cycle. However, women undergoing fertility treatment presented a lower stress-related cognitive bias when lack of joy and demands as measured by the PSQ were increased.

### Does Stress Perception and Stress-Related Cognitive Bias Change with Cycle Phases/Fertility Treatment?

During the natural cycle, significant changes in three different stress dimensions were found. While worries were higher during the pre-ovulatory phase and highest during the menstrual phase, tension, lack of joy and demands were stable throughout the cycle. As these differences are not related to estradiol levels and levels of other hormones such as progesterone or testosterone, which only differ very slightly between the menstrual and the preovulatory phase, it is not possible to provide mechanistic explanations for our findings. The lack of estradiol-related changes is also confirmed by the missing differences between various aspects of perceived stress between the end of the downregulation/preparation phase and the end of the stimulation phase, where estradiol values are highest, in women receiving fertility treatment. On this background, physical symptoms such as the bleeding or dysmenorrhea, that is, pain related to the menstrual bleeding, are likely to be responsible for differences in findings. Unfortunately, we did not evaluate any details on menstrual bleeding, for example, if heavy bleeding or pain might have increased worries during this period. As the women investigated during their menstrual cycle were not aiming for a pregnancy, frustration about the bleeding is also unlikely to explain our findings.

Also, during the menstrual phase, stress-related cognitive bias was highest, while no differences were detected in different phases of a fertility treatment. Although, it would be plausible that increased worries during the menstrual phase interfere with cognitive performance, our results on stress perception showed no association with cognitive performance. Previous research has demonstrated that cycle-related differences are not consistently found across several consecutive cycles (Leeners et al., 2017). Also, the lack of differences during the two time points of fertility treatment supports that changes in the estradiol levels alone are unlikely to induce changes in stress-related cognitive performance. Our findings are in contrast with previous results, which showed an association between estradiol and stress perception (Goldstein, 2005; Goldstein et al., 2010; Albert et al., 2015); however, due to the small sample sizes the reliability of these fMRI studies is questionable (Button et al., 2013; Turner et al., 2018). In addition to the above-mentioned misinterpretation of findings, dissimilarities in stress measurement may also account for the differences. Also, methodological aspects, for example, learning effects or the phenomenon of “Regression to the mean” might have influenced absolute test values and might consequently have reduced differences between consecutive measurements. While several available studies focus on the role of stress in the onset of psychiatric disease (Glover et al., 2012; Zoladz and Diamond, 2013), our study investigates subjective stress perception and stress-related cognitive bias.

### Are Subjectively Perceived Stress and Stress-Related Cognitive Bias Measures Different in Women During Their Menstrual Cycle and During Fertility Treatment?

Although infertility is well-known to be associated with stress (Cousineau and Domar, 2007; Gameiro et al., 2013; Rockliff et al., 2014; Pasch et al., 2016), our findings show no significant differences in the perceived stress level as well as the cognitive bias test between women receiving fertility treatment and women in their natural cycle. While the later measurements of the CBT may be influenced though learning effects and the phenomenon of “Regression to the mean,” these effects cannot explain the lack of differences in the first measurement of the series. About 15–20% of the couples receiving ART need psychosocial support (Boivin, 2002); however, the great majority succeeds to face diagnosis and treatment of infertility without additional support. This notion is sustained by our findings. Previous research has shown that although specific psychological factors associated with infertility-related distress can be identified, there are strong individual differences (Rockliff et al., 2014). Also, the concepts to provide psychological support in different centers offering fertility medicine vary greatly. In our department, two fertility specialists with a psychological education provide support for couples when needed and wanted. The lack of increased stress in the women investigated in the present study may also be explained by the fact, that infertile women experience psychological stressors as stronger burden than medical procedures such as oocyte pick-up, collection of blood samples, or anesthesia (van Balen et al., 1996; Hammarberg et al., 2001). The feeling of helplessness and waiting times, that is, phases where nothing can be done to improve chances for pregnancy are particularly stressful. While differences between women in their natural cycle and infertile women might be greater in the context of a diagnosis of infertility, shortly after treatment failure or during the often prolonged waiting times, our results have been collected during fertility treatment, that is, a very active phase, where the possibility of a pregnancy is experienced as high and positive feelings might consequently be stronger than in other phases. During this period, women are, moreover, intensively supported by doctors and nurse. This support in combination with the fact that IVF is associated with the best available chances to achieve a pregnancy may help to reduce the stress levels that are generally associated with infertility. Reduction of psychological burden in phases of high frequency contacts with health care providers has also been reported in the context of other diseases (Leeners et al., 2008). Our results on the subjective perception of stress as well as in the CBT as an objective test, support the notion that the medical procedures are not associated with increased stress levels. Also, the increasingly open discussion about infertility, along with an increasing easiness to receive support from women or couples in similar situations through the internet and a rising awareness that psychological support is an important component in successful fertility treatment, might help to reduce infertility-related stress (Boivin, 2002; Gameiro et al., 2013).

### Is There an Association Between Stress Perception and Stress-Related Cognitive Bias?

The subjectively experienced stress and stress-related cognitive bias did not correlate in women with a natural cycle. Research on stress is challenged by the absence of objective criteria to measure individual stress (Nesse et al., 2010). A lack of correlation between subjective and objective stress measures has been previously described and might also explain the lack of correlation between our PSQ and CBT findings. Nonetheless, our results confirm that stress is multidimensional and stress research should consequently combine subjective and objective as well as functional tests to collect the full picture of stress experience in study participants.

Interestingly, women undergoing fertility treatment presented a lower cognitive bias when lack of joy and demands as measured by the PSQ were increased. While we have no explanation for why the lack of joy is associated with decreased cognitive bias, adapting to higher demands might result in increased concentration, and consequently allows to reduce cognitive bias.

### Is there any Association Between Estradiol Levels and Stress Perception or the Cognitive Bias?

Currently, the role of estrogen on the response to psychosocial stress is only partly understood. In our study, higher levels of estradiol, as occurring in fertility treatments, were associated with increased cognitive bias; however, the effect size was minute. In contrast, no significant associations between estradiol levels and stress as represented by the scores in the PSQ or the CBT could be demonstrated during the natural cycle. A previous study showed that even though small changes of cognitive parameters in relation to hormonal changes can be detected in a first cycle, a comparison with a consecutive cycle shows that such changes cannot be reliably reproduced (Leeners et al., 2017). Therefore, we expect that only strongly elevated estradiol levels, as occurring in a fertility treatment, may exhibit an association with cognitive bias. A dose-dependent effect of E2 has also been described in other studies (Holmes et al., 2002; Barker and Galea, 2010). However, as stress-related cognitive performance did not differ significantly between the two time points of fertility treatment, the association between strongly elevated estradiol levels and CBT results may hint at a lack of clinical relevance. An analysis of 259 women throughout two menstrual cycles showed an association between estradiol levels and stress (Wactawski-Wende et al., 2009; Schliep et al., 2015). However, although the actual desire for a pregnancy and known gynecological problems were exclusion factors for study participation, the suspicion of a fertility problem as a motivating factor to participate in the study was not taken in consideration. As women with reproductive dysfunction showed higher stress levels and low estradiol levels may be the result of impaired follicular maturation, the association between estradiol levels and stress levels might eventually have been biased by fertility problems.

Previous studies support the role of ovarian hormones as a mediating factor for the effect of psychosocial stress (Glover et al., 2012; Albert et al., 2015). Estrogen has been shown, for example, to modulate the brain response to negatively valanced images or negative emotional information (Goldstein, 2005; Andreano and Cahill, 2010; Merz et al., 2012). As the evaluated cognitive test represents reactions to time stress, our findings for high estradiol levels seem to support such a role. While previous research has demonstrated associations between high estradiol levels and an attenuation of negative mood response to psychosocial stress, that is, increased vulnerability in phases of low estradiol levels (Albert et al., 2015), our results show increased cognitive bias when estradiol levels are very high. As estradiol has been described to attenuate sympathetic and HPA axis activity to stress (Putnam et al., 2005; Kajantie and Phillips, 2006), these findings cannot be explained by an effect of estradiol on the systemic stress reaction. Other research demonstrated an association between high estradiol levels with greater hippocampal activity during psychosocial stress in normally cycling premenopausal women (Albert et al., 2015). It is possible that such activities to process stress, that is, increased brain activities in certain regions interfere with cognitive performance. Estradiol receptors are located in a number of brain areas, including but not limited to regions important for the autonomic, hormonal, and cognitive-emotional response to psychosocial stress (Love et al., 2010). Therefore, it is difficult to evaluate the complex interactions between estradiol and stress processing with their impact on cognitive bias. As heart rate responses to stress seem to be greater at high estradiol levels, which are in line with our findings (Kirschbaum et al., 1996), estradiol seems to have opposite effects on different physiological stress reactions.

### Strength and Limitations

A great strength of this study is the combination of serial hormonal measurements with a questionnaire evaluating subjectively perceived stress and with stress-related cognitive bias. A comparison of data collected in natural cycles to those collected in fertility treatments in which estradiol levels increase far beyond the cyclic maximum represents a natural model to evaluate associations between stress and high estradiol levels.

However, important methodological aspects question the reliability of the associations described above. First, the association between self-reported measures and hormonal values is subject to random fluctuation. In contrast to the subjective stress measure, cognitive bias as a systematic functional evaluation is likely to be more reliable but may be influenced through learning effects. Also, the phenomenon of “Regression to the Mean” (Senn, 2011) will likely result in a steady reduction of extreme values, so that the last measurements in a series may show smaller differences than the first measurements. A strong correlation between changes in CBT between the two time points and the CBT results in the first measurement (*r* = 0.35) may indicate such a “Regression to the mean.” In regard to the two aforementioned limitations, we cannot fully exclude a complex interaction of limitations and factual differences.

Self-report questionnaires as the PSQ in our study may under-report the true level of distress since patients may feign emotional well-being because of social desirability or to appear psychologically healthy toward fertility specialists. However, our study participants were currently undergoing fertility treatment, that is, the decision to initiate treatment was already taken, so that such bias can be excluded. As perceived stress does not necessarily correlate to stress hormone levels (Faresjö et al., 2013; Gerber et al., 2013) it would have been beneficial to also add such stress measures to our study. Although, any woman receiving fertility treatment and sufficiently mastering the German language was invited for study participation, selection bias because of women feeling sufficiently relaxed to agree to the additional burden of study participation cannot be excluded. However, this additional burden was also relevant for women during their menstrual cycle.

The focus of our research was to evaluate associations between estradiol levels and stress. Therefore, we included women during fertility treatments irrespective of whether they were in a first or consecutive treatment cycle. Although stressors in first and later treatment cycles may be different, for example, fear of side effects in a first treatment cycle and fear of treatment failure in a later cycle, such difference should not influence our results as we evaluate associations between estradiol levels and stress or associations between perceived stress and cognitive bias. Future work should differentiate between stress related to the fertility treatment and other stress at each timepoint.

Estradiol-related effects are part of a complex network determining experiences and reactions to psychosocial stress in women; future studies should not only include further hormones such as progesterone or testosterone but also life-time positive and negative eventually traumatic experiences, personality factors, physical and mental diseases or chronic stress (Fliege et al., 2001) as well as physical and psychological factors related to the actual menstrual cycle.

## Conclusion

The presented findings support the notion that only some aspects of subjective stress perception correlate with stress-related functions. Although infertility is known to be associated with increased psychological burden, such stress seems not to be present in all phases of fertility treatment. Cycle phases correlate only in some parameters and different phases of fertility treatment do not correlate with subjectively perceived stress and stress-related cognitive bias. Only strong changes of estradiol levels were weakly associated with stress-related cognitive bias, but not with subjective experienced stress. While methodological aspects such as learning effects potentially resulting in reduction of cognitive bias or the phenomenon of “Regression to the mean” may limit the impact of the presented findings, our research emphasizes the multidimensional character of stress, and the necessity to adjust stress research to the complex nature of stress perception and processing. Also, research on the psychological burden of infertility should take into consideration that stress may vary during the different phases of fertility treatment.

## Ethics Statement

This study was carried out in accordance with the recommendations of the Swiss regimentation for studies involving human subjects as approved by the Cantonal ethics committee of Zurich, Switzerland with written informed consent from all subjects. All subjects gave written informed consent in accordance with the Declaration of Helsinki. The protocol was approved by the Cantonal ethics committee of Zurich.

## Author Contributions

BL, TK, and FI designed this study. BL, TK, CS, and YZ collected data. LS and KS performed hormonal analysis. KG and FI organized the database. BL and FI analyzed the data. BL wrote the first draft of the manuscript. TK and FI improved the first version of the manuscript. ET, TM, and ME critically revised the first version of the manuscript. All authors contributed to manuscript conception, manuscript revision, read and approved the submitted version.

### Conflict of Interest Statement

The authors declare that the research was conducted in the absence of any commercial or financial relationships that could be construed as a potential conflict of interest.

## Abbreviations

ART: Assisted reproductive techniques
CBT: Cognitive bias test
E2: Estradiol
IVF: *In Vitro* fertilization
PSQ: Perceived Stress Questionnaire.
